# Quality of life and healthcare costs of patients with allergic respiratory diseases: a cross-sectional study

**DOI:** 10.1007/s10198-023-01598-3

**Published:** 2023-07-07

**Authors:** Vivienne Hillerich, Frederik Valbert, Silke Neusser, Oliver Pfaar, Ludger Klimek, Annette Sperl, Thomas Werfel, Eckard Hamelmann, Cordula Riederer, Stefanie Wobbe-Ribinski, Anja Neumann, Jürgen Wasem, Janine Biermann-Stallwitz

**Affiliations:** 1 Institute for Healthcare Management and Research, University Duisburg-Essen, Essen, Germany; 2 Department of Otorhinolaryngology, Head and Neck Surgery, Section of Rhinology and Allergy, University Hospital Marburg, Philipps-Universität Marburg, Marburg, Germany; 3 Center for Rhinology and Allergology, Wiesbaden, Germany; 4 Clinic for Dermatology, Allergology and Venerology, Hannover Medical School, Hanover, Germany; 5 Department for Pediatrics, Children’s Center Bethel, University Bielefeld, Bielefeld, Germany; 6 Department of Health Services Research, DAK-Gesundheit, Hamburg, Germany

**Keywords:** Asthma, Rhinitis, Health Economics, Health-Related Quality of Life, Healthcare Costs, I10, I13, I18, I19

## Abstract

**Background:**

Allergic rhinitis (AR) and allergic asthma (AA) are chronic respiratory diseases that represent a global health problem. One aim of this study was to analyze the Health-related Quality of Life (HRQoL) of the patients in order to identify statistically significant influencing factors that determine HRQoL. Another aim was to assess and analyze data on cost-of-illness from a statutory health insurance perspective.

**Methods:**

The EQ-5D-5L was used to evaluate the patients’ HRQoL. To identify the factors influencing the HRQoL, a multinomial logistic regression analysis was conducted using groups based on the EQ-5D-5L index value as dependent variable. Routine data were analyzed to determine total healthcare costs.

**Results:**

The average EQ-5D-5L index was 0.85 (SD 0.20). A high age, the amount of disease costs, low internal health-related control beliefs and high ozone exposure in the residential area were found to be statistically significant influencing factors for a low HRQoL, whereas low age, male sex and a good possibility to avoid the allergens were found to be statistically significant factors influencing a high HRQoL. On average, the study participants incurred annual costs of €3072 (SD: 3485), of which €699 (SD: 743) could be assigned to allergic respiratory diseases.

**Conclusions:**

Overall, the patients in the VerSITA study showed a high level of HRQoL. The identified influencing factors can be used as starting points for improving the HRQoL of patients with allergic respiratory diseases. From the perspective of a statutory health insurance, per person expenditures for allergic respiratory diseases are rather low.

## Background

Allergic rhinitis (AR) and allergic asthma (AA) are chronic respiratory diseases that represent a global health problem [1, 2]. In Germany, 15% of the adult population suffer from AR as well as 10% of the children [3]. For AA 5% of the adults and 10% of the children are affected [4]. Both of these chronic respiratory diseases are known to have a negative effect on a person’s Health-related Quality of Life (HRQoL) [5–9]. Apart from the classic physical symptoms like sneezing attacks, rhinorrhoe, nose itching and difficult nasal breathing AR can also cause fatigue, irritability, anxiety, attention difficulties, learning disabilities, sexual dysfunction and depression [10–12]. Consecutively, AR can impair social functioning by restricting everyday activities [12]. AA is characterized by sudden attacks of dyspnoea associated with other respiratory symptoms such as cough, chest tightness and sputum production [4, 13]. Although an HRQoL reduction appears to be low in well-controlled asthma, the disease has the potential to place an enormous burden on the daily lives of minors and adults [5, 12, 14]. The fact that AA is often associated with comorbidities—including mental disorders such as depression – can have an additional negative impact on HRQoL as well as on the success of controlling symptoms therapeutically [15, 16]. Since AR and AA often occur together respectively 25% of AR patients develop AA within the first ten years of the disease [1], the impairment of HRQoL supposably increases.

In addition to the clinical implication and the affection of HRQoL, both diseases have a significant social economic impact [2]. Several cost of illness studies detected major expenses due to use of healthcare resources as well as loss of productivity which manifests itself as both absenteeism and presenteeism [2]. Moreover, the costs of illness are important due to the high prevalence. Nevertheless, current health economic data for the German healthcare system regarding AR and AA are rare [17, 18].

The aim of this study was to assess the HRQoL of the population as well as to examine for certain influencing factors that determine if the HRQoL is on a high, intermediate or low level, which could provide information on how the HRQoL of patients with AR or AA could be improved. In addition, data on cost-of-illness of German patients with AR and AA was analyzed from a statutory health insurance perspective to obtain information about the economic impact of both diseases to the statutory health insurance.

## Methods

### Study design and questionnaire

A retrospective cross-sectional study called “Analysis of the care situation with regard to the specific immunotherapy in cases of allergic respiratory diseases” (VerSITA) was performed in collaboration with the Institute for Healthcare Management and Research of the University Duisburg-Essen, the statutory health insurance DAK-Gesundheit (DAK) and representatives of the German Allergy League (“Deutsche AllergieLiga”). It included patients insured by the DAK who suffered from AR and/or AA.

Patients were asked to fill in a self-administered questionnaire which included information on socio-demographic, psycho-social and clinical patient characteristics as well as the Euroqol—5 Dimensions–5 Levels (EQ-5D-5L) questionnaire, which is a generic standardized instrument to measure HRQoL.

In addition, routine data of the DAK were submitted with the permission of the study participants. For the analyses routine data and survey data were merged. Further information on how the study was conducted, how participants were selected and a description of the VerSITA questionnaire can be found elsewhere [19].

For the analysis, a classification into disease groups (“only allergic rhinitis” (oAR), “only allergic asthma” (oAA) and “patients with both diseases” (AR & AA)) and a description of patient characteristics was performed. In the cost-of-illness analysis, the entire study population (adults as well as minors) was included; in the analysis of HRQoL, only patients who had validly completed the EQ-5D-5L and were older than 18 years were included since the German version of the EQ-5D-5L used here has been assessed for adults only.

Statistical analysis was performed using IBM SPSS Statistics (version 25) and Microsoft Excel (version 2016).

### Methods of the cost-of-illness analysis

The cost-of-illness analysis from a statutory health insurance perspective was performed using primary and routine data from the study participants in the VerSITA trial. Routine data covered the period 01/01/2014–30/06/2018. The total costs include outpatient physician contacts, hospitalization, rehabilitation, medication, sick leave benefits, remedies and therapeutic appliances.

The costs of statutory health insurance are predominantly calculated on the basis of actual expenditure. To avoid the economic relevance of diseases being determined by the income of the persons concerned, the real costs of sick leave benefits are not reported here. Instead, every day sick leave that causes costs for the statutory health insurance is priced with the quotient of the expenditures for sick leave benefits of all German statutory health insurances and a total number of sick leave benefit days of the respective year [20–29]. In addition, the average lost revenues were added to this due to the fact that the insured do not pay contributions to health insurance while on sick leave [30].

To determine annual costs, the costs of the 4.5 years were divided by the respective number of days under observation (days insured by DAK) and then multiplied by 365.25 (taking leap years into account).

If an outpatient physician contact was coded with a confirmed International Statistical Classification of Diseases and Related Health Problems (ICD) diagnosis of J30.1 or J30.2 or J30.3 or J30.4, it was interpreted as a contact due to only AR. A confirmed diagnosis of J45.0 or J45.8 led to the assignment to only AA. If confirmed ICD codes for both conditions appear within a single outpatient treatment case, it was not classified as described above but categorized as due to AR as well as AA. The same approach was used for cost assignments regarding sick leave benefits. For hospitalization and rehabilitation, only diagnoses coded as "main diagnosis" were considered. The identification of medication costs due to allergen immunotherapy (AIT) (V01AA02, V01AA03, V01AA04, V01AA05, V01AA10, V01AA11, and V01AA20) or asthma medication was done via Anatomical Therapeutic Chemical codes.

### Variables in the analysis of HRQoL

The EQ-5D-5L self-administered questionnaire was used to collect the patients’ present HRQoL. This instrument consists of a descriptive system containing five questions, which inquire if there are no, slight, moderate, severe or extreme problems (depicted by the numbers 1 to 5) in the five dimensions mobility, self-care, usual activities, pain/physical discomfort and anxiety/depression. From the resulting individual health state (consisting of five numbers depicting the strength of problems in each dimension, also called descriptive system) the EQ-5D-5L index value representing the individual HRQoL in a range from –0.661 (worst possible health, worse than death respectively) to 1.000 (best possible health) is generated. The value set used to generate the EQ-5D-5L index value with preferences of the German population was developed by Ludwig et al. [31].

Moreover, an additional part of the EQ-5D-5L is the Euroqol-Visual Analogue Scale (EQ-VAS), a 15 cm long scale labelled from 0 to 100 in which participants are supposed to subjectively judge their present health state. Both the EQ-5D-5L index value as well as the EQ-VAS value was used in the analysis.

The socio-demographic variables collected were sex, age (years as well as different age groups), highest school degree and highest completed professional training, smoking status, migration background and native speaker as well as the living situation (living alone vs. living in community). In addition, data derived from the provided first three digits of the postcode of each patient were linked with publicly available data from the German Federal Statistical Office on population density [32], the number of households in need of social welfare [33] and the amount of building land prices [34]. Connecting these information was possible by using the respective official municipality codes to differentiate between dependent and district cities. This resulted in the following variables: settlement structure depending on the population density (rural area for < 200/urban area for 200–999/agglomeration area for 1,000 or more inhabitants per km^2^, definition taken from Heineberg et al. 2017 [35]), the quote of social welfare (percentage of persons in need to claim social welfare of the total German population) and the attractiveness of the residential area (indicated by the average costs of building land in € per m^2^). Also, data from the German Environment Agency on the level of environmental pollution was used and the most recent annual mean values of the airway-relevant pollutants fine dust (PM10), nitrogen dioxide (NO2) and ozone (O3) were applied [36–39]. The geographic coordinates of the respective measuring stations as well as of the postal code areas were used to assign the air pollutant values (in µg/m^3^) of the three closest measuring stations to the respective postal code area of the patient [40, 41]. A weighting was then applied, incorporating the data from these three measuring stations with a weight reciprocal to the distance in kilometers. This approach takes into account that there can be no hard geographical lines in the dispersion of air pollutants, which means that air quality in a given area is influenced from several directions—essentially depending on the distance.

As clinical parameters, the amount of allergens was surveyed as well as the possibility of allergen avoidance. Also, the severity of symptoms was ascertained using two disease-specific questionnaires: The Asthma Control Test (ACT) for patients with AA and the Rhinitis Total Symptom Score (RTSS) for patients with AR. The value of the RTSS increases with the severity of symptoms (0–12 points) [42]; a higher ACT corresponds to a better asthma control (5–25 points) [43]. In addition, to evaluate the patients’ conviction of control over their personal health (health-related control conviction) the test FEGK (Fragebogen zur Erfassung gesundheitsbezogener Kontrollüberzeugungen) by D. Ferring (2003) was applied [44]. It consists of a total of ten questions, from which a score indicating the strength of the internality (conviction to be able to control and influence his or her personal health) or externality (conviction of the contrary) is derived. The respective score was converted into a categorical variable using the median of the respective score as the cut-off value for high or low internality/externality (48 for internality and 64 for externality).

Furthermore, routine data on costs from a statutory health insurance perspective were used as an indicator of allergy severity and severity of comorbidities. Since allergic respiratory diseases are almost exclusively treated on an outpatient basis, the costs due to outpatient physician contacts, that could be associated with the allergic respiratory diseases, were considered as a further indicator of allergy severity (specific outpatient disease costs). The sum of the remaining healthcare costs was used as a simplified indicator for comorbidities (remaining healthcare costs) including costs for hospitalization, rehabilitation, medication, sick leave benefits, remedies, therapeutic appliances as well as the remaining proportion of outpatient disease costs that are not related to allergic respiratory diseases.

### Statistical analysis of HRQoL

Sociodemographic and clinical data of the total study population as well as of each disease group (oAR, oAA and AR & AA) where described using relative and absolute frequencies. To verify significant differences between the groups, the Chi^2^-test or the Mann–Whitney U test was used depending on the scale level. In all statistical analyses values of *p* < 0.05 were considered as statistically significant.

The HRQoL of the patients was described by using all parts of the EQ-5D-5L, including the descriptive system, the EQ-5D-5L index value as well as the EQ-VAS value.

To discover factors that determine the HRQoL of the VerSITA participants a multinomial logistic regression was performed using the EQ-5D-5L index value as the dependent variable by dividing the study population into groups with high, intermediate and low HRQoL. To achieve preferably equal-sized groups, the 33th percentile was used. The group with intermediate HRQoL was taken as a reference category. This way, four logistic analyses were conducted using each HRQoL group with regard to the different disease groups as well as the overall study population as a dependent variable.

All variables mentioned above were included in the analysis, except for the variable number of allergens due to problems of interpretability as well as lots of missing values. To achieve a sufficiently great number of cases in each category the independent variable highest school degree was transformed into the three groups high, medium and low level of education. For the same reason, the categories very good and good possibility to avoid allergens were merged.

To rule out multi-collinearity a bivariate collinearity analysis was performed and variables with a significant Pearson-collinearity value of > 7.0 were excluded.

Microsoft Excel (version 2008) was used to process raw data and create the independent variables. All further analyses were conducted with the Software IBM SPSS Statistics (version 27).

## Results

### Results regarding healthcare costs

A sample of 21,901 insured patients was drawn and 2,505 responded with analyzable data and a valid consent form (another 358 responded without valid informed consent or generally non-analyzable questionnaires) which corresponds to a response rate of 11.4% (concerning only valid responses). Due to the inclusion of underaged persons and study participants with invalid EQ-5D-5L forms, healthcare cost data were available on all 2505 study participants with analyzable data and valid informed consent (while 2128 study participants remained for the HRQoL analysis).

The mean age in the whole study population was 48.3 years (SD: 20.6), 46.6 years (SD: 21.2) in the oAR group, 49.7 years (SD: 21.7) in the oAA group, and 48 years (SD: 19.3) in the AR & AA group. 67.1% of the total study participants were female (63.6% in the oAR group, 63.2% in the oAA group, and 72.3% in the AR & AA group). The allergens to which most study participants were allergic were tree pollen (75.8%), grass pollen (72.8%) and dust mites (57.7%). Most study participants reacted to three or more allergens (63.1%). A more detailed descriptive of the study population has already been published elsewhere [45].

Table 1 shows a description of the annual healthcare costs in € from statutory health insurance perspective. There are a few implausible values observable (for example, costs due to AA in the oAR group). A possible explanation for this can be found in the discussion section. On average, the study participants incurred annual costs of €3,072 (SD: 3,485), of which €699 (SD: 743) could be assigned to allergic respiratory diseases.

**Table 1:** Annual healthcare costs in € from a statutory health insurance perspective

	oAR (*n* = 560)	oAA (*n* = 893)	AR & AA (*n* = 1052)	Overall (*n* = 2505)
	Annual health costs in € (MV (SD))			
**Outpatient physician contacts**	731.48 (622.62)	950.02 (1143.60)	932.53 (704.88)	893.82 (876.71)
of which due to AR	145.22 (122.47)	73.47 (132.88)	97.36 (121.06)	99.54 (128.46)
of which due to AA	17.73 (72.57)	116.84 (146.54)	86.53 (134.42)	81.96 (133.32)
of which due to AR & AA	21.6 (70.94)	106.81 (144.82)	115.67 (143.70)	91.48 (136.69)
**Hospitalization**	553.64 (1212.14)	931.83 (1830.51)	780.42 (1739.34)	783.70 (1676.64)
of which due to AR	0	0	0.07 (2.31)	0.03 (1.50)
of which due to AA	0	9.27 (69.20)	5.21 (43.47)	5.49 (50.11)
**Rehabilitation**	32.12 (131.09)	52.71 (189.17)	57.48 (216.56)	50.11 (190.70)
of which due to AR	3.24 (38.55)	0.32 (9.50)	0	0.84 (19.12)
of which due to AA	0	3.11 (38.05)	6.12 (70.63)	3.68 (51.14)
**Medication**	803.40 (1826.85)	1140.08 (2111.96)	928.16 (1241.33)	975.82 (1731.46)
of which for AIT	277.85 (376.96)	190.45 (344.95)	235.55 (354.81)	228.93 (357.82)
of which for asthma medication	24.16 (83.34)	247.69 (639.11)	200.97 (448.47)	178.10 (488.59)
**Sick leave benefits**	89.65 (678.89)	94.27 (798.09)	142.61 (891.29)	113.54 (814.76)
of which due to AR	0	0	0	0
of which due to AA	0	0	21.16 (395.38)	8.88 (256.37)
**Remedies**	115.25 (277.43)	153.24 (388.95)	155.42 (359.69)	145.66 (354.46)
**Therapeutic appliances**	92.22 (249.08)	111.61 (306.72)	116.89 (459.36)	109.49 (368.81)
**Total**	2417.76 (3011.38)	3433.78 (3805.22)	3113.50 (3387.87)	3072.14 (3484.71)
of which can be assigned to the allergic respiratory diseases	489.80 (429.97)	747.96 (815.72)	768.64 (788.19)	698.93 (742.79)

*n* = number of patients, *MV = *mean value, *SD* = standard deviation, *oAR* = only allergic rhinitis, *oAA* = only allergic asthma, *AR* & *AA* = allergic rhinitis and allergic asthma. Minor discrepancies between total costs and the sum of the separate items are due to rounding

A relatively high proportion of expenditures for outpatient physician contacts (€273 of €894, 31%) and for medication (€407 of €976, 42%) was triggered by allergic respiratory diseases. In both cases, the highest proportion of expenditures due to allergic respiratory diseases can be found in the study group AR & AA (23% and 47%, respectively). In the other categories, allergic respiratory diseases as a cost trigger played only a minor role. Here, costs due to, presumably severe, comorbidities are predominant. Overall, 9% of the expenditures for rehabilitation (€5 of 50€), 8% of the expenditures for sick leave benefits (€9 of €114), and 1% of the expenditures for hospitalization (€6 of €784) could be assigned to allergic respiratory diseases. Again, differences can be seen between the study groups; for example, only in group AR & AA costs were incurred because of sick leave benefits. The evaluated routine data did not allow an assignment of costs to allergic respiratory diseases in the categories of remedies and therapeutic appliances.

Overall, 23% of the average statutory health insurance expenditure in the study population could be attributed to allergic respiratory diseases.

In addition, there were costs that are usually not reimbursed or only partially reimbursed by the statutory health insurance (for example for over-the-counter medication or alternative medicine). They were consequently not included in the perspective of statutory health insurance. Nevertheless, it should be noted that 62% of the study participants reported expenditures on over-the-counter medications 12 months prior to the survey and 41% reported the use of alternative medicine due to their allergic respiratory diseases.

It should also be noted that the relative proportion of costs due to allergic respiratory diseases as a percentage of total costs is clearly higher among minors (41.5%). On the one hand, this is related to their typically lower total costs. On the other hand, there are increased costs attributable to allergic respiratory diseases, since 80.5% of minors received AIT during the routine data period (vs. 50.6% of adults).

### Patient characteristics within the HRQoL-analysis

After the reduction of invalid questionnaires due to an incomplete EQ-5D-5L as well as questionnaires of minors for evaluating the HRQoL, a study population of a total of 2128 adult patients with allergic respiratory diseases resulted in the HRQoL-analysis, of which 477 patients were assigned to the group oAR, 743 patients to the group oAA, and 908 patients to the group AR & AA. Table 2 lists the sociodemographic as well as clinical characteristics. It also shows for each variable whether the patients in the respective disease group differ statistically significant from the rest.

**Table 2:** Sociodemographic and clinical characteristics of the study population of the HRQoL analysis

Variable	oAR (*n* = 477)	oAA (*n* = 743)	AR & AA (*n* = 908)	Overall (*n* = 2128)
**Age (years) **(MV, SD)	51.24 (18.068)	54.64 (17.396)**	51.64 (15.907)**	52.59 (16.994)
**Sex female** (*n*, %)	311 (65.2)**	500 (67.3)	677 (74.6)**	1488 (69.9)
**German citizenship **(*n*, %)	446 (93.5)	683 (91.9)	846 (93.2)	1975 (92.8)
**Age group** (*n*,%)	**	**	**	
18–29 years	79 (16.6)	86 (11.6)	96 (10.6)	261 (12.3)
30–44 years	88 (18.4)	108 (14.5)	187 (20.6)	383 (18.0)
45–64 years	180 (37.7)	299 (40.2)	426 (46.9)	905 (42.5)
≥ 65 years	130 (27.3)	250 (33.6)	199 (21.9)	579 (27.2)
**Highest school degree** (*n*, %):	**	**	**	
No specification	4 (0.8)	16 (2.2)	16 (1.8)	36 (1.7)
Still in school education	1 (0.2)	1 (0.1)	3 (0.3)	5 (0.2)
No graduation	2 (0.4)	8 (1.1)	2 (0.2)	12 (0.6)
Lower secondary qualification	67 (14.0)	189 (25.4)	127 (14.0)	383 (18.0)
Higher secondary qualification	169 (35.4)	260 (35.0)	365 (40.2)	794 (37.3)
Higher education entrance qualification	234 (49.1)	269 (36.2)	395 (43.5)	898 (42.2)
**Highest completed professional training** (*n*, %):	**	**		
No specification	5 (1.0)	22 (3.0)	11 (1.2)	38 (1.8)
Still in training	24 (5.0)	28 (3.8)	28 (3.1)	80 (3.8)
None	15 (3.1)	40 (5.4)	22 (2.4)	77 (3.6)
Apprenticeship	191 (40.0)	320 (43.1)	380 (41.9)	891 (41.9)
Technical college	92 (19.3)	121 (16.3)	187 (20.6)	400 (18.8)
University of applied science	41 (8.6)	57 (7.7)	78 (8.6)	176 (8.3)
University	90 (18.9)	97 (13.1)	140 (15.4)	327 (15.4)
Other	19 (4.0)	58 (7.8)	62 (6.8)	139 (6.5)
**Smoking status **(*n*, %):				
No specification	1 (0.2)	5 (0.7)	7 (0.8)	13 (0.6)
Non-smoker	287 (60.2)	441 (59.4)	535 (58.9)	1263 (59.4)
Former smoker	142 (29.8)	229 (30.8)	280 (30.8)	651 (30.6)
Active smoker	47 (9.9)	68 (9.2)	86 (9.5)	201 (9.4)
**Migration background** (*n*, %)*:	44 (9.3)	66 (9.1)	72 (8.0)	182 (8.7)
**Native speaker **(*n*, %)*:	454 (95.2)	706 (95.0)	865 (95.3)	2025 (95.2)
Number of allergens*	**	**	**	
One allergen	112 (24.3)	125 (18.8)	75 (8.3)	312 (15.5)
Two allergens	131 (28.4)	155 (23.3)	144 (15.9)	430 (21.4)
Three or more allergens	218 (47.3)	384 (57.8)	667 (73.5)	1269 (63.1)
Possibility of allergen avoidance*	**	**	**	
Very good	3 (0.7)	36 (5.1)	14 (1.6)	53 (2.5)
Good	41 (9.2)	142 (20.3)	102 (11.4)	285 (13.4)
Moderate	101 (22.5)	226 (32.3)	298 (33.4)	625 (29.4)
Poor	118 (26.3)	134 (19.1)	232 (26.0)	484 (22.7)
Very poor	129 (28.8)	95 (13.6)	177 (19.8)	401 (19.7)
No experiences	56 (12.5)	67 (9.0)	69 (7.7)	192 (9.4)
**RTSS-Score**				
*n*	449	−	848	−
MV (SD)	7.163 (2.704)	−	7.205 (2.723)	−
Modus	8	−	6	−
**ACT-Score**				
*n*	−	697	855	–
MV (SD)	−	18.971 (5.053)	19.051 (5.031)	–
Modus	−	25	25	–
Internality*	**			
High	450 (97.4)	690 (95.7)	849 (94.6)	1989 (95.6)
Low	12 (2.6)	31 (4.3)	48 (5.4)	91 (4.4)
Externality*	**		**	
High	153 (33.4)	273 (38.2)	364 (40.9)	790 (38.3)
Low	305 (66.6)	442 (61.8)	527 (59.1)	1274 (61.7)
**Indicators of allergy severity/severity of comorbidities** (MV [SD] in €)	**	**	**	
Spedific outpatient disease costs	189.11 (142.16)	291.98 (192.04)	295.08 (185.89)	270.25 (184.47)
Remaining healthcare costs***	2375.70 (3077.76)	3238.56 (3798.07)	2919.03 (3421.87)	2908.80 (3499.57)

*n* = number of patients, *MV* = mean value, *SD* = standard deviation, *oAR* = only allergic rhinitis, *oAA* = only allergic asthma, *AR*&*AA* = allergic rhinitis and allergic asthma. *In the case of migration background, 32 study participants could not be evaluated; In the case of a native speaker, 27 study participants could not be evaluated; In case of number of allergens, 117 study participants could not be evaluated; in case of the possibility of allergen avoidance, 88 study participants could not be evaluated; in case of internality, 48 study participants could not be evaluated, in case of externality, 64 study participants could not be evaluated; for these variables % = valid %. **Significant difference from the rest of the study population according to the chi-square test (significance level: < 0.05). ***including costs for hospitalization, rehabilitation, medication, sick leave benefits, remedies, therapeutic appliances as well as the remaining proportion of outpatient disease costs that are not related to allergic respiratory diseases

The average age was 53 years. The group of 18–29 year-old patients represented the smallest proportion of just over 12%.

In terms of the educational and professional background of all 2128 patients, it is noticeable that 42% had a higher education entrance qualification (“allgemeine Hochschulreife/Fachhochschulreife”) and most patients, again 42%, had completed an apprenticeship (“Lehre”). A total of 77 patients (3.6%) stated that they had not completed any professional training with 12 patients (0.6%) not having a graduation.

Nearly 60% of all patients were non-smokers. The proportion of active smokers was 9.4% overall.

8.7% of all patients had a migration background, meaning either they, themselves or their parents were not born in Germany. Around 95% of all patients were native speakers.

Almost two-thirds (63%) reacted to at least three different allergens. The proportion of monoallergic patients was highest in the oAR group with 24.3%.

Only 2.5% stated that they were able to avoid the allergens causing the disease very well. The ability to avoid allergens was most often moderate (29.4%), but patients having oAR mostly stated that they could avoid allergens very poorly (28.8%).

Patients suffering from oAR reported an average RTSS of 7.2, which corresponds to a moderate symptom severity. They most frequently documented an average RTTS of 8, which was 2 points higher compared to patients suffering from AR & AA.

The ACT averaged 19 points. However, excellent asthma control, calculated as 25 points, was most frequently among patients with oAA as well as patients with AR & AA.

Regarding the health-related control conviction measured by the FEGK, it is noticeable that only 91 patients (4.4%) had a low internal control conviction. In contrast, more than 95% were rather convinced that they could actively influence their own health. In terms of externality, 62% showed a low external control conviction, while 38% were rather convinced that they could not control their own health.

Specific outpatient disease costs averaged 270.25€ per patient (SD 184.47€, minimum 0€ to maximum 1,638.26€) in this specific analysis subpopulation. With an average of 2,908.80€ (SD 3,499.57, minimum 39.21€ to maximum 35,347.77€), the remaining healthcare costs respectively the costs for comorbidities were substantially higher.

One-fifth of all patients stated that they lived alone and not in a community with a partner, family or friends. In terms of settlement structure, in the entire study population most patients (40.5%) lived in an urbanized area; around 30% of each disease group lived in either rural or agglomeration areas. The quote of social welfare averaged 7.1% overall (SD 3.38%); the average cost of building land was € 369.55 per m^2^ (SD 492.76). The most recent annual mean values of air pollutants were 16.99 µg/m^3^ for PM10 (year limit 40 µg/m^3^ [46]), NO2 26.97 µg/m^3^ (year limit 40 µg/m^3^ [47]) and O3 50.13 µg/m^3^ (year limit 120 µg/m^3^ [48]).

### Results regarding HRQoL

The HRQoL of the entire study population averaged 0.85 using the EQ-5D-5L index value; the EQ-VAS value averaged 73 (see Table 3). Patients with oAR had the highest average HRQoL (0.887 respectively 77.56). Compared to this, patients with oAA and patients with AR & AA had lower scores (0.842 and 70.72, respectively, 0.836 and 71.92).

**Table 3:** Descriptive statistic of the EQ-5D-5L Index and the EQ-VAS

		Min–max	MV (SD)	Median
**Overall** (*n* = 2,128)	EQ-5D-5L	−0.378 – 1.000	0.85 (0.20)	0.913
	EQ-VAS	5 – 100	72.76 (16.86)	75
**oAR** (*n* = 477)	EQ-5D-5L*	−0.250 – 1.000	0.887 (0.173)	0.943
	EQ-VAS*	8 – 100	77.56 (15.35)	80
**oAA** (*n* = 743)	EQ-5D-5L	−0.378 – 1.000	0.842 (0.201)	0.907
	EQ-VAS*	5 – 100	70.73 (17.03)	75
**AR & AA** (*n* = 908)	EQ-5D-5L*	−0.335 – 1.000	0.836 (0.210)	0.907
	EQ-VAS*	10 – 100	71.92 (16.91)	75

*n* = number of patients, *MV* = mean value, *SD* = standard deviation, *oAR* = only allergic rhinitis, *oAA* = only allergic asthma, *AR* & *AA* = allergic rhinitis and allergic asthma. *Significant difference from the rest of the study population according to the chi^2^ test (significance level: < 0.05)

Looking more closely at the individual dimensions of the EQ-5D-5L it is noticable that in four of five dimensions most patients did not declare any problems (see Table 4). Only in the category pain or physical discomfort most patients had at least slight problems in all disease groups. The dimension anxiety was the second most frequent issue in all groups—the highest proportion of having at least slight problems was in patients with AR & AA (45.7%). This was followed by problems with usual activities and mobility with similarly high proportions (32.7% and 32.29%, respectively). Extreme problems in at least one dimension were rarely seen with an average of around 0.5%, where problems in the category pain were the most likely.

**Table 4:** Absolute and relative response frequencies in the dimensions of the EQ-5D-5L referring to the total sample and the individual disease groups

	EQ-5D-5L dimension	No problems *n* (%)	Slight problems *n* (%)	Moderate problems *n* (%)	Severe problems *n* (%)	Extreme problems *n* (%)	At least slight problems *n* (%)
**Overall** (*n* = 2,128)	Mobility	1439 (67.6)	367 (17.2)	218 (10.2)	99 (4.7)	5 (0.2)	689 (32.3)
	Self care	2005 (94.2)	69 (3.2)	28 (1.3)	20 (0.9)	6 (0.3)	123 (5.7)
	Usual activities	1434 (67.4)	419 (19.7)	204 (9.6)	63 (3.0)	8 (0.4)	694 (32.7)
	Pain or physical discomfort	607 (28.5)	858 (40.3)	479 (22.5)	162 (7.6)	22 (1.0)	1521 (71.4)
	Anxiety or depression	1232 (57.9)	576 (27.1)	221 (10.4)	83 (3.9)	16 (0.8)	896 (42.2)
**oAR** (*n* = 477)	Mobility	359 (75.3)	68 (14.3)	32 (6.7)	17 (3.6)	1 (0.2)	118 (24.7)
	Self care	464 (97.3)	6 (1.3)	2 (0.4)	5 (1.0)	0 (0.0)	13 (2.7)
	Usual activities	383 (80.3)	58 (12.2)	26 (5.5)	8 (1.7)	2 (0.4)	94 (19.7)
	Pain or physical discomfort	167 (35.0)	204 (42.8)	79 (16.6)	22 (4.6)	5 (1.0)	310 (65.0)
	Anxiety or depression	307 (64.4)	119 (24.9)	39 (8.2)	10 (2.1)	2 (0.4)	170 (35.6)
**oAA** (*n* = 743)	Mobility	470 (63.3)	133 (17.9)	98 (13.2)	40 (5.4)	2 (0.3)	273 (36.7)
	Self care	687 (92.5)	32 (4.3)	12 (1.6)	9 (1.2)	3 (0.4)	56 (7.5)
	usual activities	465 (62.6)	165 (22.2)	88 (11.8)	24 (3.2)	1 (0.1)	278 (37.4)
	Pain or physical discomfort	217 (29.2)	274 (36.9)	188 (25.3)	58 (7.8)	6 (0.8)	526 (70.8)
	Anxiety or depression	432 (58.1)	196 (26.4)	77 (10.4)	31 (4.2)	7 (0.9)	311 (41.9)
**AR & AA** (*n* = 908)	Mobility	610 (67.2)	166 (18.3)	88 (9.7)	42 (4.6)	2 (0.2)	298 (32.8)
	Self care	854 (94.1)	31 (3.4)	14 (1.5)	6 (0.7)	3 (0.3)	54 (5.9)
	Usual activities	586 (64.5)	196 (21.6)	90 (9.9)	31 (3.4)	5 (0.6)	322 (35.5)
	Pain or physical discomfort	223 (24.6)	380 (41.9)	212 (23.3)	82 (9.0)	11 (1.2)	685 (75.4)
	Anxiety or depression	493 (54.3)	261 (28.7)	105 (11.6)	42 (4.6)	7 (0.8)	415 (45.7)

*n* = number of patients, *oAR* = only allergic rhinitis, *oAA* = only allergic asthma, *AR* &* AA* = allergic rhinitis and allergic asthma

The results of the multinomial logistic regression based on the EQ-5D-5L index value for the total study population are presented in detail in Table 5; the allocation of HRQoL groups for the dependent variable of each regression is shown in Table 6.

**Table 5:** Results of the multinomial logistic regression based on the EQ-5D-5L index value regarding influencing factors on HRQoL based on the EQ-5D-5L for the total study population (*n* = 2128)

Variable	Regression coefficient B	Standard error	Wald	df	Sig	Exp(B)
Low HRQoL according to EQ-5D-5L (reference: intermediate HRQoL)						
Constant	−2.000	1.163	2.958	1	0.085	
Remaining healthcare costs	0.120	0.019	40.047	1	0.000	1.127
Specific outpatient costs of disease	0.810	0.336	5.823	1	0.016	2.248
Quote of social welfare	0.007	0.025	0.069	1	0.793	1.007
Attractiveness of residential area	0.000	0.000	2.057	1	0.152	1.000
O3	0.038	0.015	6.729	1	0.009	1.038
PM10	0.003	0.032	0.007	1	0.934	1.003
NO2	0.013	0.010	1.726	1	0.189	1.013
Age group*^1^						
18–29 years	0.025	0.281	0.008	1	0.929	1.025
30–44 years	−0.135	0.205	0.433	1	0.510	0.873
45–64 years	0.365	0.149	6.016	1	0.014	1.441
Sex (male)*^2^	0.131	0.137	0.910	1	0.340	1.140
No migration background*^3^	−0.289	0.203	2.036	1	0.154	0.749
Level of education*^4^						
High level of education	−0.264	0.209	1.598	1	0.206	0.768
Medium level of education	−0.113	0.170	0.443	1	0.506	0.893
Highest completed professional training*^5^						
University	−0.705	0.302	5.464	1	0.019	0.494
University of applied science	−0.484	0.324	2.224	1	0.136	0.616
technical college	−0.380	0.257	2.182	1	0.140	0.684
Apprenticeship	−0.253	0.236	1.152	1	0.283	0.776
still in professional training	−0.276	0.471	0.345	1	0.557	0.758
no completed professional training	0.639	0.389	2.703	1	0.100	1.894
Smoking status*^6^						
Non-smoker	−0.227	0.208	1.187	1	0.276	0.797
Former smoker	0.028	0.219	0.016	1	0.899	1.028
Settlement structure*^7^						
Rural area	−0.221	0.274	0.654	1	0.419	0.802
Urbanized area	−0.053	0.225	0.056	1	0.813	0.948
Living in community*^8^	−0.378	0.144	6.894	1	0.009	0.685
Possibility of allergen avoidance*^9^						
Good possibilities to avoid allergen	−0.329	0.254	1.683	1	0.195	0.720
Moderate possibilities to avoid allergen	−0.033	0.224	0.022	1	0.883	0.968
Bad possibilities to avoid allergen	0.018	0.230	0.006	1	0.939	1.018
Very bad possibilities to avoid allergen	−0.063	0.240	0.069	1	0.793	0.939
Health-related control conviction*^10^						
Low level of externality	−0.337	0.121	7.753	1	0.005	0.714
Low level of internality	1.281	0.293	19.070	1	0.000	3.599
High HRQoL according to EQ-5D-5L (reference: intermediate HRQoL)						
Constant	1.274	1.187	1.151	1	0.283	
Remaining healthcare costs	−0.114	0.032	12.584	1	0.000	0.892
Specific outpatient costs of disease	−0.454	0.395	1.315	1	0.251	0.635
Quote of social welfare	−0.027	0.025	1.125	1	0.289	0.974
Attractiveness of the residential area	0.000	0.000	0.143	1	0.705	1.000
O3	−0.022	0.015	2.081	1	0.149	0.978
PM10	−0.036	0.032	1.261	1	0.261	0.964
NO2	−0.002	0.010	0.041	1	0.839	0.998
Age group*^1^						
18–29 years	0.944	0.244	14.932	1	0.000	2.571
30–44 years	0.784	0.198	15.710	1	0.000	2.190
45–64 years	0.104	0.173	0.360	1	0.548	1.109
Sex (male)*^2^	0.289	0.132	4.754	1	0.029	1.335
No migration background*^3^	0.264	0.227	1.354	1	0.245	1.302
level of education*^4^						
High level of education	0.033	0.229	0.020	1	0.886	1.033
Medium level of education	−0.050	0.200	0.062	1	0.803	0.951
Highest completed professional training*^5^						
University	0.010	0.305	0.001	1	0.974	1.010
University of applied science	0.001	0.334	0.000	1	0.999	1.001
Technical college	−0.133	0.291	0.208	1	0.648	0.876
Apprenticeship	−0.133	0.269	0.244	1	0.621	0.875
Still in professional training	−0.043	0.404	0.011	1	0.915	0.958
No completed professional training	0.321	0.441	0.529	1	0.467	1.378
Smoking status*^6^						
Non-smoker	0.074	0.209	0.125	1	0.724	1.077
Former smoker	0.022	0.227	0.010	1	0.922	1.022
Settlement structure*^7^						
Rural area	−0.114	0.274	0.172	1	0.678	0.893
Urbanized area	0.036	0.225	0.026	1	0.872	1.037
Living in community*^8^	0.021	0.161	0.017	1	0.896	1.021
Possibility of allergen avoidance*^9^						
Good possibilities to avoid allergen	0.326	0.236	1.912	1	0.167	1.386
Moderate possibilities to avoid allergen	−0.395	0.225	3.087	1	0.079	0.674
Bad possibilities to avoid allergen	−0.681	0.235	8.397	1	0.004	0.506
Very bad possibilities to avoid allergen	−0.477	0.240	3.951	1	0.047	0.620
Health related control conviction*^10^						
Low level of externality	0.181	0.131	1.898	1	0.168	1.198
Low level of internality	0.174	0.402	0.188	1	0.665	1.190

Nagelkerkes-R-square 23.9%, *n* = 1848 (13.2% missing cases due to missing values); Reference categories for categorical variables: *^1^ ≥ 65 years, *^2^female, *^3^migration background, *^4^low level of education, *^5^other completed professional training, *^6^active smoker, *^7^agglomeration area, *^8^living alone, *^9^no experience in avoiding an allergen, *^10^high level of externality / internality. *O*3 = ozone, *PM*10 = fine dust, *NO*2 = nitrogen dioxide

**Table 6:** Allocation of the HRQoL-Groups including amount and index range

EQ-5D-5L (*n*, index range)				
	Overall (*n* = 2128)	oAR (*n* = 477)	oAA (*n* = 743)	AR & AA (*n* = 908)
High HRQoL	560 (0.944–1.000)	155 (0.959–1.000)	200 (0.944–1.000)	203 (0.944–1.000)
Intermediate HRQoL (reference)	918 (0.861 – 0.943)	163 (0.895 – 0.958)	295 (0.853 – 0.943)	401 (0.861 – 0.943)
Low HRQoL	650 (−0.378 – 0.860)	159 (−0.250 – 0.894)	248 (−0.378 – 0.852)	304 (−0.335 – 0.860)

*n* = number of patients, *oAR* = only allergic rhinitis, *oAA* = only allergic asthma, *AR* & *AA* = allergic rhinitis and allergic asthma

When the variable was significant with a positive regression coefficient, there was an increased likelihood that the patient was classified in the group with higher HRQoL compared to the reference group having intermediate HRQoL; when the sign of the regression coefficient was negative, there was a corresponding decreased likelihood. This model explained 23.9% of the variance in HRQoL differences.

Comparing patients with low HRQoL and patients with intermediate HRQoL (reference group) the level of remaining healthcare costs (*B* = 0.120) as well as the level of allergy-specific outpatient disease costs (*B* = 0.810) were statistically significant factors influencing low HRQoL. In addition, high ambient ozone exposure (*B* = 0.038), being aged 45 to 64 years (*B* = 0.365) as well as having a low level of internality (*B* = 1.281) were statistically significant factors contributing to a low HRQoL. Having a university degree (*B* = −0.705), living in a community (*B* = −0.378), and a low level of externality (*B* = −0.337) were statistically significant protective factors against low HRQoL.

When comparing the group with high HRQoL and the reference group, the age of 18–29 years (*B* = 0.944) and 30–44 years (*B* = 0.784) as well as male sex (*B* = 0.289) were statistically significant factors influencing high HRQoL. Statistically significant factors against a high HRQoL were a high level of remaining healthcare costs (*B* = −0.114) as well as poor and very poor possibilities to avoid the allergens (*B* = −0.681 and −0.477, respectively).

Looking at the individual disease groups, there are minor differences in terms of the influencing factors (the detailed regression results can be found in the appendix).

In patients suffering from oAR (see Table 7), the level of comorbidities was a statistically significant factor associated with low HRQoL, whereas living in a community proved to be a statistically significant protective factor against low HRQoL. Similar to the overall study population, having a low level of internality was found to be a statistically significant factor influencing a low HRQoL in patients suffering from oAR. Patients suffering from oAR with high HRQoL were statistically significant characterized by at least good possibilities to avoid allergens, low PM10 exposure and a young age of 18–29 years.

With respect to the patients with oAA (see Table 8), a high ACT, meaning a good symptom control, was a statistically significant protective factor against a low HRQoL as well as a positive influencing factor for a high HRQoL. The remaining healthcare costs contributed statistically significant to a low HRQoL; the allergy-specific outpatient disease costs were shown to be a statistically significant factor against a high HRQoL. Male sex, the two younger age groups (18–29 years and 30–45 years) as well as a low level of externality were found to be statistically significant factors for a high HRQoL.

For patients in the group AR & AA high remaining healthcare costs, high ozone exposure and a low ACT turned out to be statistically significant factors indicating a low HRQoL (see Table 9). Living in a community proved to be a statistically significant protective factor against low HRQoL. Young age of 18–29 years was found to be a statistically significant factor influencing a high HRQoL.

## Discussion

The aim of this study was to evaluate the HRQoL of the patients and to identify influencing factors for a high and low HRQoL as well as to analyze the financial impact of patients with AR and AA in Germany to the statutory health insurance.

The results of the present analysis and their interpretation are affected by some limitations.

### Discussion regarding general limitations of the study

A potential bias results from the low rate of valid responses (11.4%), which was probably on the one hand due to the length of the questionnaire and the postal effort required to return it. On the other hand, it has to be mentioned that routine data is prone for miscoding. It is, therefore, possible that some of the non-responders did not react because they do not suffer from an allergic respiratory disease, even if it is coded that way. This is in line with the remarkable observation that (in a minor extent) in the group oAR costs were caused due to AA and vice versa in the group oAA due to AR. Here, the information from the patient survey on which the group allocation is based on obviously differs from the documented ICD codes in the routine data. This might be due to individual miscoding in the routine data (for example because diagnoses may be routinely coded even if they are not applicable in the individual case). This concern is in line with the general discussion on the quality of routine data [49–52]. Nonetheless, the effect of this bias on overall costs can be considered as minor and it has to be mentioned that, despite possible limitations in data quality, routine data analysis is a common method in science [53, 54]. However, the resulting study population of more than 2000 subjects is large enough to allow valid statistical conclusions to be drawn in principle.

### Discussion regarding healthcare costs

With regard to the healthcare costs used in both analyses, a period of 4.5 years was documented, so that previous costs were not recorded. However, the period is long enough to allow conclusions about the state of the patient’s illness, the existence of chronic comorbidities or to calculate average annual costs.

A small group of persons had gaps in the insurance period. Due to the approach of calculating the costs depending on the number of insured days, the impact is estimated to be very minor.

The extent of information in the routine data implies the risk that not all costs incurred due to allergic respiratory diseases could be assigned to them. For example, only a very general categorization was available for remedies, which did not allow the identification of therapies that may have been linked to an allergic respiratory disease. Since the total costs were considered, this has no effect on the sum of the costs, but the costs which can be assigned to the allergic respiratory diseases might be slightly underestimated. On the other hand, the fact that contacts with the healthcare system related to allergic respiratory diseases were attributed 100% to them may lead to an overestimation of the costs which can be assigned to the allergic respiratory diseases in the analyses of healthcare costs.

A large proportion of individuals with AIT within the routine data period were intentionally recruited for questions regarding the care situation (published elsewhere) [19, 45]. The resulting AIT rate in the VerSITA study of 54% within the routine data period is far above the amount that is estimated for Germany and leads to an overestimation of the average costs for AIT medication. This may also have increased the costs for outpatient physician care. On the other hand, AIT is considered as cost-effective [55, 56]. Thus, it can be assumed that the high AIT rate in the studies also contributes to savings.

A further overestimation of the costs could be due to the fact that the selection of study participants was based on routine data. As a result, people with allergic respiratory diseases who do not get in touch with the healthcare system for this purpose are not included.

Overall, due to the limitations mentioned above, an overestimation of the total costs for the statutory health insurance system which can be assigned to allergic respiratory diseases is to be expected within this study.

Even though the economic burden of allergic respiratory diseases on statutory health insurance is shown to be rather low, it should be considered that many drugs against AR are paid for by the insured as over-the-counter medication. Furthermore, it could be shown that almost half of the study participants used alternative medicine. Other studies have also shown that a relevant proportion of the costs of allergic respiratory diseases are indirect macroeconomic costs, for example, due to absenteeism and presentism [57, 58]. Because of the health insurance perspective in this study, these costs were not obtained here.

Since the study participants in the three disease groups differ from each other in numerous characteristics (see a more detailed description of the study population in [45]), cost differences between the groups cannot be explained here by the variable that led to the allocation into the groups (oAR, oAA and AR & AA).

A comparison of the results based on already published evidence is not very reliable. Thus, a large part of the previously published cost data on allergic respiratory diseases in Germany is at least 20 years old [18]. A study frequently cited in this context (Schramm et al. 2003, data collection March 1999 to February 2000) indicates that the costs of "moderate-to-severe atopic asthma and/or seasonal allergic rhinitis" tend to be higher [57]. This is probably explainable by a very different study design in Schramm et al. In addition, it is conceivable that healthcare reforms or expiring patents had a cost-reducing effect as well within this rather large period of time. A more recent national analysis can be found in the publication by Tesch et al. 2020 [17]. Due to the different sampling, the costs in the VerSITA study and from the publication by Tesch et al. are not reliably comparable. In VerSITA, as explained above, a selective study population was needed for analyses regarding AIT. In Tesch et al., however, AIT was not a selection criterion and was determined retrospectively [17]. Since the performance of AIT depends on characteristics such as certain comorbidities, the different selection has a strong effect on the composition of the study population. It is likely that the results of Tesch et al. are more generalizable. In addition, the procedure of operationalizing AIT in Tesch et al. differs from the procedure in the VerSITA study, which makes it difficult to compare subgroups (for example, only persons with AIT) [17]. A comparison with international literature is not appropriate here since different health care systems have potentially a high impact on the calculated costs from the perspective of healthcare insurance. Due to the differences in health care systems and the specifics of this analysis mentioned above, the comparability of the reported costs with international evidence is hardly possible.

The consideration of total costs allows an interesting comparison: statutory health insurers spent an average of €3,287 per insured person in 2018 [20]. Taking into account that this sum also includes a small amount of non-medical costs, such as administrative costs, it becomes clear that the average of the study population is very similar to the average for all statutory health insured in terms of average total costs. Overall, the calculation and descriptive analysis of costs should not be seen as the main focus of the publication, but as complementary to the analyses concerning HRQoL.

### Discussion regarding HRQoL

In the HRQoL assessment, the overall average EQ-5D-5L index value was 0.85 (SD 0.20) and the average EQ-VAS was 72.76 (SD 16.86). Compared to surveys in the German general population using the EQ-5D-5L from Grochtdreis et al. (2019), Ludwig et al. (2018) and Huber et al. (2017) the EQ-VAS value of the VerSITA population is about 3.3 EQ-VAS points lower than the population-weighted average EQ-VAS value in the general population of 76.1 [31, 59, 60]. When looking at the disease groups, it is noticeable that the patients with oAR revealed an even slightly higher HRQoL compared to the general German population in the last five years (EQ-VAS in the oAR group: 77.6). This may be related to the fact that the oAR group most frequently included patients with young age and high level of education. Also, it seems remarkable that patients with AR & AA had – compared to those with oAA—equally high (average EQ-5D-5L Index of 0.836 respectively 0.842) or even higher (average EQ-VAS of 71.92 respectively 70.73) HRQoL values. On the one hand, this result could be due to a higher proportion of patients aged 65 years or older (33.6% in the AA group compared to 21.9% in the AR & AA group) as well as more patients with low educational level (28.8% in the AA group compared to 16.3% in the AR & AA group). Another possible explanation could be that symptoms due to AA are perceived comparatively less important than symptoms due to AR. Also, this result might be related to the fact that patients with AR often develop AA in the course of life [1]. It could be plausible that, in these cases, the symptoms of the patient are predominated by AA although the diagnosis of AR is still coded in the routine data so that he or she was thereby assigned to the group with AR & AA. As one of the few studies that collected HRQoL from both patients with AR and AA similar to the VerSITA study, Chen et al. found a comparable result in 545 patients from California resulting in mean EQ-5D(-3L) index values of 0.76 for both patients with oAA and AR & AA. Patients with oAR also had a significantly higher mean score of 0.92 in Chen et al. [61].

In a national comparison, there are only a few studies of comparable size and method that have assessed the HRQoL of patients with allergic respiratory diseases using the EQ-5D-5L. Szentes et al. evaluated the HRQoL of 371 German patients with AA in a longitudinal survey using the EQ-5D-5L. Similar high mean values as in the VerSITA study of 0.84 (EQ-5D-5L index) and 67.1 (EQ-VAS) were obtained [62]. With regard to AR, there are, to our knowledge, currently no comparable German studies existing. In an international context, the results can be compared to the survey of Bousquet et al., who assessed the HRQoL of 843 patients with AR in France and evaluated an average EQ-VAS value of 75.47, which is similar to the VerSITA study [63].

In terms of the dimensions of the EQ-5D it can be seen that the frequency of a perfect health state (no problems in all five dimensions) is lower in the VerSITA population (20.6%) compared to the studies in the general population (on average 43.9% in the German general population) [31, 59, 60]. This suggests a greater limitation of HRQoL in patients with AA and/or AR. It is known that the so-called ceiling effect is prevalent in the descriptive system of EQ-5D-5L, especially in studies in the normal population [64]. However, Konnopka et al. showed in their analysis that a considerable part of the frequently observed ceiling effect in population surveys is actually due to the correct measurement of health status because the level of the EQ-5D-5L index score is strongly dependent on the amount of comorbidities or morbidity [65]. Taking these findings into account, the lower proportion of patients at VerSITA with no problems in any of the dimensions of the EQ-5D-5L compared with the general population—despite the only slightly lower mean values of EQ-VAS and EQ-5D-5L index score—does suggest greater HRQoL limitations in patients with AA and/or AR.

High healthcare costs (allergy-specific outpatient disease costs as well as remaining healthcare costs), a strong external respectively low internal health-related control conviction, high age and high ozone exposure are plausible factors influencing a low HRQoL. It seems obvious that relevant comorbidities can strongly influence the HRQoL of patients. When considering the whole study population, the amount of the specific outpatient disease costs may be an even more reliable indicator for the burden of disease of the patients than the AR and AA specific symptom scores, since the disease groups are mixed here.

In other studies, higher age has been shown to be a relevant factor limiting the HRQoL [5, 66, 67]. Ozone may increase both lung function limitation as well as the inflammatory response to an inhaled allergen in individuals with  preexisting allergic airway disease [68], which could explain the effect on HRQoL observed in this analysis.

Young age and male sex were found to be statistically significant factors influencing a higher HRQoL. Both of these factors have been shown in studies before, especially on HRQoL in the general population [5, 66, 67, 69, 70]. University education was shown to be a protective factor against low HRQoL, which fits with the findings of Burr et al. who observed in the general population significantly lower levels of poor health for persons working in simple manual occupations or agricultural laborers than for professions such as engineers, teachers and physicians that require a university degree [66]. Indirectly, these results also support the finding of a study by Blozik et al., who determined that patients with AA and low educational or occupational status had greater impairment of HRQoL [5].

In people with AA, symptom control was shown to influence HRQoL statistically significant, whereas symptom severity in AR patients assessed by RTSS did not show a statistically significant result in any regression. This could be due to the fact that only a few, and only classical clinical symptoms were captured by RTSS, meaning that effects like fatigue, impairment of cognitive function and depression in particular were not directly surveyed. Another reason could be that the HRQoL of AR patients is not significantly shaped by the classic rhinitis symptoms, but more by the identified factors (especially severe or many comorbidities and low internal control beliefs).

It should be noted that there is an increased proportion of patients in the VerSITA population who performed AIT, so the HRQoL might be increased by the fact that more patients than normally received a causally effective treatment. In addition, due to the use of self-administered questionnaires, there was a high number of missing values due to invalid or missing crosses of certain questions for lots of independent variables. Depending on the regression analysis this led to the exclusion of 13,2% to 20,4% of the cases, which is likely to result in an overall reduction of the quality measure.

All variables derived from the abbreviated postal code of patients are inherently subject to the limitation that they are less precise than they would be if all digits of the postal code would have been available.

In principle, the conclusions drawn regarding the factors influencing the HRQoL of the patients are limited by the fact that a cross-sectional study is only a momentary image, which may be influenced by the individual's mood depending on a particular day.

At the time the VerSITA study was conducted, the multinomial logistic regression method used to evaluate the EQ-5D-5L had not yet been utilized. The most comparable type of analysis is a cross-sectional study by Barth et al. who dichotomized the EQ-5D-3L Index score as the dependent variable using the 10th percentile to investigate factors influencing HRQoL in patients with juvenile arthritis and hence conducted a binary logistic regression [71]. With regard to the applied method in this paper, one has to point out that the EQ-5D-5L index instrument results from a broad condensation of a few weighted health-related components. This summarization is necessary to use the EQ-5D-5L efficiently for cost–benefit analyses and allocation decisions in the healthcare system. However, there is a risk that the complexity of the HRQoL is not sufficiently captured by only five dimensions and that important aspects of the HRQoL are missed. In addition, the EQ-5D-5L is a generic instrument that allows a broad application but does not specifically address disease-typical symptoms. This results in a reduced sensitivity regarding the HRQoL in patients with AA and/or AR. However, the EQ-5D-5L including the EQ-VAS is an established instrument and, therefore, has the advantage that corresponding results can be compared with pre-existing surveys in the normal population, especially since there are no predata regarding the HRQoL of allergic patients using the EQ-5D-5L in a comparable German population, yet.

## Conclusions

The VerSITA study provides a current and detailed report on the costs of patients with allergic respiratory diseases from the perspective of statutory health insurance. It was shown that from the perspective of statutory health insurance costs per person due to allergic respiratory diseases are rather low. However, expenditures outside the statutory health insurance system are very common for these diseases.

Overall, it can be concluded that the adult patients in the VerSITA study basically showed a high level of HRQoL with patients having oAR reaching slightly higher levels than patients with oAA or patients with both diagnoses. Possible startings points for improving the HRQoL of patients with allergic respiratory diseases should be derived from the identified influencing factors. If the HRQoL of patients with allergic respiratory diseases should be improved or optimized, one can, according to the results of the present study, on the one hand start with the health-related control beliefs and, for example, as a physician or therapist try to encourage a more internal control conviction. On the other hand, the improvement of or attention to the air quality in the patient's environment can be beneficial (especially ozone and fine dust emissions). Equally important, especially in patients with AA, is the optimal control of symptoms with medication and, in general, the best possible treatment of comorbidities in all patients.

## Data Availability

For reasons of privacy and contractual obligations, the data analyzed here cannot be made publicly available.

## Appendix

See Tables 7, 8 and 9.

**Table 7:** Influencing factors on HRQoL based on the EQ-5D-5L regarding the group oAR

Variable	Regression coefficient B		Standard error	Wald	df	Sig	Exp(B)
Low HRQoL according to EQ-5D-5L (reference: intermediate HRQoL)							
Constant	3.595		2.753	1.705	1	0.192	
Remaining healthcare costs	0.115		0.047	5.944	1	0.015	1.122
Specific outpatient costs of disease	−0.311		1.025	0.092	1	0.762	0.733
Quote of social welfare	0.061		0.058	1.073	1	0.300	1.062
Attractiveness of residential area	−0.001		0.000	2.493	1	0.114	0.999
O3	−0.027		0.035	0.606	1	0.436	0.973
PM10	−0.082		0.071	1.338	1	0.247	0.921
NO2	0.018		0.023	0.621	1	0.431	1.018
RTSS	−0.041		0.053	0.611	1	0.434	0.960
18–29 years	−0.142		0.555	0.066	1	0.797	0.867
30–44 years	−0.605		0.473	1.638	1	0.201	0.546
45–64 years	0.248		0.362	0.468	1	0.494	1.281
Sex (male)*^2^	0.160		0.298	0.287	1	0.592	1.173
No migration background*^3^	−0.231		0.466	0.246	1	0.620	0.794
High level of education	−0.177		0.495	0.128	1	0.721	0.838
Medium level of education	0.099		0.417	0.056	1	0.812	1.104
University	0.195		0.808	0.058	1	0.810	1.215
University of applied science	1.097		0.883	1.545	1	0.214	2.996
Technical college	0.436		0.750	0.337	1	0.562	1.546
Apprenticeship	0.695		0.734	0.897	1	0.344	2.004
Still in professional training	0.435		1.164	0.139	1	0.709	1.545
No completed professional training	0.614		1.067	0.331	1	0.565	1.847
Non-smoker	−0.482		0.473	1.038	1	0.308	0.617
Former smoker	−0.409		0.505	0.655	1	0.418	0.664
Rural area	0.143		0.637	0.051	1	0.822	1.154
Urbanized area	0.071		0.550	0.016	1	0.898	1.073
Living in community*^8^	−0.855		0.357	5.736	1	0.017	0.425
Good possibilities to avoid allergen	−0.777		0.627	1.538	1	0.215	0.460
Moderate possibilities to avoid allergen	−0.926		0.516	3.221	1	0.073	0.396
Bad possibilities to avoid allergen	−0.608		0.499	1.485	1	0.223	0.545
Very bad possibilities to avoid allergen	−0.442		0.503	0.771	1	0.380	0.643
Low level of externality	−0.531		0.288	3.407	1	0.065	0.588
Low level of internality	2.371		1.145	4.286	1	0.038	10.705
High HRQoL according to EQ-5D-5L (reference: intermediate HRQoL)							
Constant		1.985	2.680	0.549	1	0.459	
Remaining healthcare costs		−0.051	0.062	0.685	1	0.408	0.950
Specific outpatient costs of disease		−1.487	1.146	1.685	1	0.194	0.226
Quote of social welfare		−0.022	0.058	0.150	1	0.698	0.978
Attractiveness of residential area		0.000	0.000	0.090	1	0.764	1.000
O3		−0.008	0.034	0.059	1	0.808	0.992
PM10		−0.168	0.069	6.018	1	0.014	0.845
NO2		0.044	0.023	3.712	1	0.054	1.045
RTSS		−0.052	0.052	0.997	1	0.318	0.949
18–29 years		1.077	0.524	4.227	1	0.040	2.935
30–44 years		0.823	0.450	3.337	1	0.068	2.276
45–64 years		0.576	0.415	1.930	1	0.165	1.779
Sex (male)*^2^		0.231	0.287	0.649	1	0.420	1.260
No migration background*^3^		0.376	0.484	0.602	1	0.438	1.456
High level of education		0.266	0.549	0.235	1	0.628	1.305
Medium level of education		0.502	0.491	1.045	1	0.307	1.652
University		1.005	0.844	1.418	1	0.234	2.732
University of applied science		1.712	0.917	3.484	1	0.062	5.542
Technical college		0.390	0.838	0.216	1	0.642	1.477
Apprenticeship		0.335	0.806	0.172	1	0.678	1.397
Still in professional training		1.005	0.991	1.028	1	0.311	2.731
No completed professional training		1.027	1.070	0.921	1	0.337	2.794
Non-smoker		0.212	0.467	0.206	1	0.650	1.236
Former smoker		0.358	0.510	0.494	1	0.482	1.431
Rural area		−0.335	0.618	0.293	1	0.588	0.716
Urbanized area		−0.247	0.526	0.221	1	0.638	0.781
Living in community*^8^		−0.001	0.391	0.000	1	0.999	0.999
Good possibilities to avoid allergen		−0.786	0.585	1.805	1	0.179	0.455
Moderate possibilities to avoid allergen		−1.144	0.504	5.161	1	0.023	0.318
Poor possibilities to avoid allergen		−1.426	0.506	7.951	1	0.005	0.240
Very poor possibilities to avoid allergen		−1.331	0.506	6.916	1	0.009	0.264
Low level of externality		−0.030	0.304	0.010	1	0.921	0.970
Low level of internality		−0.394	1.513	0.068	1	0.795	0.675

Nagelkerkes-R-square 23.9%, *n* = 1848 (13.2% missing cases due to missing values); Reference categories for categorical variables: *^1^ ≥65 years, *^2^female, *^3^migration background, *^4^low level of education, *^5^other completed professional training, *^6^active smoker, *^7^agglomeration area, *****^**8**^living alone, *^9^no experience in avoiding an allergen, *****^**10**^high level of externality/ internality. O3 = ozone, PM10 = fine dust, NO2 = nitrogen dioxide

**Table 8:** Influencing factors on HRQoL based on the EQ-5D-5L regarding the group oAA

Variable	Regression coefficient B	Standard error	Wald	df	Sig	Exp(B)
Low HRQoL according to EQ-5D-5L (reference: intermediate HRQoL)						
Constant	0.949	2.225	0.182	1	0.670	
Remaining healthcare costs	0.094	0.030	9.827	1	0.002	1.098
Specific outpatient costs of disease	0.076	0.597	0.016	1	0.898	1.079
Quote of social welfare	–0.087	0.049	3.089	1	0.079	0.917
Attractiveness of residential area	0.000	0.000	0.791	1	0.374	1.000
O3	0.052	0.029	3.344	1	0.067	1.054
PM10	0.024	0.059	0.173	1	0.678	1.025
NO2	0.014	0.018	0.568	1	0.451	1.014
ACT	−0.148	0.024	37.833	1	0.000	0.862
18–29 years	−0195	0544	0.128	1	0.721	0.823
30–44 years	−0.039	0.415	0.009	1	0.925	0.962
45–64 years	0.463	0.275	2.836	1	0.092	1.589
Sex (male) *^2^	0.476	0.248	3.701	1	0.054	1.610
No migration background*^3^	−0.483	0.364	1.760	1	0.185	0.617
High level of education	0.085	0.391	0.048	1	0.827	1.089
Medium level of education	0.013	0.299	0.002	1	0.966	1.013
University	−0.687	0.566	1.472	1	0.225	0.503
University of applied science	−1.203	0.622	3.734	1	0.053	0.300
Technical college	−0.326	0.468	0.485	1	0.486	0.722
Apprenticeship	−0.123	0.413	0.089	1	0.765	0.884
Still in professional training	0.608	0.784	0.601	1	0.438	1.837
No completed professional training	0.645	0.618	1.089	1	0.297	1.907
Non-smoker	−0.389	0.403	0.936	1	0.333	0.677
Former smoker	−0.391	0.419	0.872	1	0.350	0.676
Rural area	−0.952	0.510	3.476	1	0.062	0.386
Urbanized area	−0.495	0.410	1.457	1	0.227	0.610
Living in community*^8^	−0.069	0.276	0.063	1	0.801	0.933
Good possibilities to avoid allergen	−0.391	0.426	0.841	1	0.359	0.676
Moderate possibilities to avoid allergen	0.016	0.396	0.002	1	0.969	1.016
Bad possibilities to avoid allergen	−0.436	0.426	1.049	1	0.306	0.646
Very bad possibilities to avoid allergen	−0.136	0.464	0.086	1	0.770	0.873
Low level of externality	−0.376	0.230	2.674	1	0.102	0.687
Low level of internality	0.893	0.535	2.785	1	0.095	2.443
High HRQoL according to EQ-5D-5L (reference: intermediate HRQoL)						
Constant	−0.082	2.356	0.001	1	0.972	
Remaining healthcare costs	−0.070	0.045	2.443	1	0.118	0.932
Specific outpatient costs of disease	−2.143	0.726	8.724	1	0.003	0.117
Quote of social welfare	–0.034	0.047	0.513	1	0.474	0.967
Attractiveness of residential area	0.000	0.000	0.094	1	0.760	1.000
O3	−0.022	0.030	0.521	1	0.470	0.979
PM10	−0.015	0.060	0.061	1	0.805	0.985
NO2	−0.014	0.019	0.567	1	0.451	0.986
ACT	0.062	0.030	4.338	1	0.037	1.064
18–29 years	0.914	0.452	4.087	1	0.043	2.495
30–44 years	0.869	0.374	5.413	1	0.020	2.385
45–64 years	0.124	0.298	0.173	1	0.677	1.132
Sex (male) *^2^	0.520	0.244	4.528	1	0.033	1.681
No migration background*^3^	0.028	0.400	0.005	1	0.944	1.028
High level of education	−0.117	0.397	0.087	1	0.768	0.890
Medium level of education	−0.505	0.332	2.320	1	0.128	0.603
University	0.009	0.533	0.000	1	0.987	1.009
University of applied science	0.197	0.586	0.112	1	0.737	1.217
Technical college	−0.142	0.512	0.077	1	0.782	0.868
Apprenticeship	0.020	0.453	0.002	1	0.965	1.020
Still in professional training	–0.342	0.734	0.218	1	0.641	0.710
No completed professional training	0.050	0.696	0.005	1	0.943	1.051
Non-smoker	0.457	0.418	1.195	1	0.274	1.579
Former smoker	0.105	0.446	0.056	1	0.813	1.111
Rural area	−0.380	0.516	0.541	1	0.462	0.684
Urbanized area	−0.055	0.420	0.017	1	0.895	0.946
Living in community*^8^	−0.027	0.286	0.009	1	0.924	0.973
Good possibilities to avoid allergen	0.819	0.426	3.688	1	0.055	2.268
Moderate possibilities to avoid allergen	0.415	0.432	0.923	1	0.337	1.514
Poor possibilities to avoid allergen	−0.114	0.469	0.060	1	0.807	0.892
Very poor possibilities to avoid allergen	0.014	0.518	0.001	1	0.979	1.014
Low level of externality	0.502	0.247	4.145	1	0.042	1.652
Low level of internality	0.370	0.695	0.284	1	0.594	1.448

Nagelkerkes-R-square 38.2%, *n* = 593 (20.2% missing due to missing values); Reference categories for categorical variables: *^1^ ≥ 65 years, *^2^female, *^3^migration background, *^4^low level of education, *^5^other completed professional training, *^6^active smoker, *^7^agglomeration area, *****^**8**^living alone, *^9^no experience in avoiding an allergen, *****^**10**^high level of externality/ internality. O3 = ozone, PM10 = fine dust, NO2 = nitrogen dioxide

**Table 9:** Influencing factors on HRQoL based on the EQ-5D-5L regarding the group AR & AA

Variable	Regression coefficient B		Standard error		Wald	df	Sig	Exp(B)
Low HRQoL according to EQ-5D-5L (reference: intermediate HRQoL)								
Constant	−3.310		1.859		3.170	1	0.075	
Remaining healthcare costs	0.179		0.036		24.405	1	0.000	1.196
Specific outpatient costs of disease	0.186		0.555		0.112	1	0.737	1.205
Quote of social welfare	0.002		0.039		0.004	1	0.952	1.002
Attractiveness of residential area	0.000		0.000		0.667	1	0.414	1.000
O3	0.055		0.022		6.199	1	0.013	1.056
PM10	0.033		0.051		0.419	1	0.518	1.033
NO2	0.015		0.016		0.825	1	0.364	1.015
RTSS	0.013		0.035		0.131	1	0.717	1.013
ACT	−0.054		0.019		8.181	1	0.004	0.948
18–29 years	0.104		0.456		0.052	1	0.819	1.110
30–44 years	−0.270		0.322		0.705	1	0.401	0.763
45–64 years	0.396		0.255		2.425	1	0.119	1.487
Sex (male) *^2^	−0.129		0.227		0.322	1	0.571	0.879
No migration background*^3^	−0.203		0.343		0.350	1	0.554	0.816
High level of education	0.261		0.345		0.569	1	0.451	1.298
Medium level of education	0.320		0.300		1.138	1	0.286	1.377
University	−0.511		0.491		1.085	1	0.297	0.600
University of applied science	−0.035		0.502		0.005	1	0.944	0.965
Technical college	−0.449		0.424		1.125	1	0.289	0.638
Apprenticeship	−0.071		0.395		0.032	1	0.858	0.932
Still in professional training	−0.173		0.740		0.055	1	0.815	0.841
No completed professional training	0.995		0.715		1.935	1	0.164	2.705
Non-smoker	0.138		0.335		0.170	1	0.680	1.148
Former smoker	0.423		0.352		1.439	1	0.230	1.526
Rural area	−0.315		0.439		0.516	1	0.472	0.730
Urbanized area	−0.197		0.359		0.301	1	0.583	0.821
Living in community*^8^	−0.456		0.229		3.967	1	0.046	0.634
Good possibilities to avoid allergen	0.200		0.465		0.184	1	0.668	1.221
Moderate possibilities to avoid allergen	0.040		0.407		0.010	1	0.922	1.041
Bad possibilities to avoid allergen	−0.028		0.421		0.004	1	0.947	0.972
Very bad possibilities to avoid allergen	−0.098		0.435		0.051	1	0.822	0.906
Low level of externality	−0.126		0.193		0.424	1	0.515	0.882
Low level of internality	0.798		0.419		3.638	1	0.056	2.222
High HRQoL according to EQ-5D-5L (reference: intermediate HRQoL)								
Constant	1.032	2.087		0.245		1	0.621	
Remaining healthcare costs	−0.172	0.071		5.811		1	0.016	0.842
Specific outpatient costs of disease	0.537	0.682		0.619		1	0.432	1.710
Quote of social welfare	−0.022	0.044		0.239		1	0.625	0.979
Attractiveness of residential area	0.001	0.000		3.523		1	0.061	1.001
O3	−0.054	0.027		4.007		1	0.045	0.947
PM10	−0.001	0.059		0.001		1	0.980	0.999
NO2	−0.020	0.019		1.140		1	0.286	0.980
RTSS	−0.066	0.041		2.574		1	0.109	0.936
ACT	0.079	0.025		10.122		1	0.001	1.082
18–29 years	1.037	0.447		5.383		1	0.020	2.821
30–44 years	0.409	0.353		1.342		1	0.247	1.506
45–64 years	−0.149	0.326		0.210		1	0.647	0.861
Sex (male) *^2^	0.150	0.239		0.394		1	0.530	1.162
No migration background*^3^	0.136	0.438		0.096		1	0.756	1.146
High level of education	0.640	0.445		2.068		1	0.150	1.897
Medium level of education	0.549	0.405		1.840		1	0.175	1.732
University	−0.661	0.532		1.543		1	0.214	0.516
University of applied science	−0.504	0.592		0.726		1	0.394	0.604
Technical college	−0.448	0.495		0.820		1	0.365	0.639
Apprenticeship	−0.234	0.449		0.271		1	0.603	0.792
Still in professional training	−0.318	0.701		0.207		1	0.649	0.727
No completed professional training	−0.631	0.979		0.415		1	0.519	0.532
Non-smoker	−0.029	0.354		0.007		1	0.934	0.971
Former smoker	−0.044	0.384		0.013		1	0.910	0.957
Rural area	0.313	0.488		0.410		1	0.522	1.367
Urbanized area	0.351	0.393		0.800		1	0.371	1.421
Living in community*^8^	0.112	0.285		0.153		1	0.695	1.118
Good possibilities to avoid allergen	0.272	0.472		0.331		1	0.565	1.312
Moderate possibilities to avoid allergen	−0.734	0.433		2.876		1	0.090	0.480
Poor possibilities to avoid allergen	−0.739	0.446		2.744		1	0.098	0.478
Very poor possibilities to avoid allergen	−0.268	0.457		0.343		1	0.558	0.765
Low level of externality	0.185	0.226		0.673		1	0.412	1.204
Low level of internality	0.654	0.577		1.285		1	0.257	1.923

Nagelkerkes-R-square 30.3%, *n* = 723 (20.4% missing due to missing values); Reference categories for categorical variables: *^1^ ≥ 65 years, *^2^female, *^3^migration background, *^4^low level of education, *^5^other completed professional training, *^6^active smoker, *^7^agglomeration area, *****^**8**^living alone, *^9^no experience in avoiding an allergen, *****^**10**^high level of externality/ internality.O3 = ozone, PM10 = fine dust, NO2 = nitrogen dioxide

## Abbreviations

AA: Allergic asthma
ACT: Asthma Control Test
AIT: Allergen immunotherapy
AR: Allergic rhinitis
AR&AA: Group: patients with allergic rhinitis and allergic asthma
oAA: Group: patients with only allergic asthma
oAR: Group: patients with only allergic rhinitis
B: Regression coefficient
DAK: DAK-Gesundheit
DRKS: German Clinical Trials Register
EQ-5D-3L: EuroQol-5 Dimension-3 Level
EQ-5D-5L: EuroQol-5 Dimensionen-5 Level
EQ-VAS: EuroQol Visual Analogue Scale
FEGK: Fragebogen zur Erfassung gesundheitsbezogener Kontrollüberzeugungen
HRQoL: Health-related Quality of Life
ICD: International Statistical Classification of Diseases and Related Health Problems
MV: Mean value
n: Number of cases
NO2: Nitrogen dioxide
O3: Ozone
PM10: Fine dust
RTSS: Rhinoconjunctivitis Total Symptom Score
SD: Standard deviation
VerSITA: Analysis of the care situation with regard to the specific immunotherapy in cases of allergic respiratory diseases

## References

[1] Bousquet J, Khaltaev N, Cruz AA, Denburg J, Fokkens WJ, Togias A, Zuberbier T, Baena-Cagnani CE, Canonica GW, van Weel C, Agache I, t-Khaled, N.A., Bachert, C. (2008). allergic rhinitis and its impact on asthma (ARIA) 2008 update (in collaboration with the World Health Organization, GA(2)LEN and AllerGen). Allergy.

[2] Bousquet J, Van Cauwenberge P, Khaltaev N, Aria Workshop G, World Health O (2001). Allergic rhinitis and its impact on asthma. J. Allergy Clin. Immunol..

[3] Langen U, Schmitz R, Steppuhn H (2013). Häufigkeit allergischer erkrankungen in deutschland - ergebnisse der studie zur gesundheit erwachsener in deutschland (DEGS1). Bundesgesundheitsbl..

[4] Buhl R, Berdel D, CriØe C-P, Gillissen A, Kardos P, Kroegel C, Leupold W, Lindemann H, Magnussen H, Nowak D, Pfeiffer-Kascha D, Rabe K, Rolke M (2006). S2k-Leitlinie zur diagnostik und therapie von patienten mit asthma. Pneumologie.

[5] Blozik E, Demmer I, Kochen MM, Koschack J, Niebling W, Himmel W, Scherer M (2009). Gesundheitsbezogene lebensqualität bei asthmapatienten in der hausarztpraxis. Dtsch. Med. Wochenschr..

[6] Johnson, J.: Einfluss der saisonalen allergischen Rhinitis auf die Lebensqualität, die Tagesschläfrigkeit und objektive Schlafparameter. http://archiv.ub.uni-heidelberg.de/volltextserver/7805/1/diss07_124.pdf (2006). Accessed 2 January 2021

[7] Canonica G, Mullol J, Pradalier A, Didier A (2008). Patient perceptions of allergic rhinitis and quality of life: findings from a survey conducted in europe and the United States. World Allergy Organ. J..

[8] Price D, Scadding G, Ryan D, Bachert C, Canonica G, Mullol J, Klimek L, Pitman R, Acaster S, Murray R, Bousquet J (2015). The hidden burden of adult allergic rhinitis: UK healthcare resource utilisation survey. Clin. Translational Allergy.

[9] McDonald VM, Hiles SA, Jones KA, Clark VL, Yorke J (2018). Health-related quality of life burden in severe asthma. Med J Aust..

[10] Biedermann T, Renz H, Heppt W, Röcken M (2016). Allergologie.

[11] Bachert C, Borchard U, Wedi B, Klimek L, Rasp G, Riechelmann H, Schultze-Werninghaus G, Wahn U, Ring J (2003). Allergische rhinokonjunktivitis - leitlinie der deutschen gesellschaft für allergologie und klinische immunologie (DGAI). Allergo J..

[12] Pawankar R, Canonica GW, Holgate ST, Lockey RF,Blaiss MS (2013) White Book on Allergy - Update 2013. World Allergy Organization.

[13] Herold G (2020) Innere Medizin. Köln: Gerd Herold.

[14] Poethko-Müller C, Thamm M, Thamm R (2018). Heuschnupfen und asthma bei kindern und jugendlichen in deutschland – querschnittergebnisse aus KiGGS welle 2 und trends. J. Health Monit..

[15] Boulet L-P, Boulay M-È (2011). Asthma related comorbidities. Expert Rev. Respir. Med..

[16] Strine TW, Ford ES, Balluz L, Chapman DP, Mokdad AH (2004). Risk behaviors and health-related quality of life among adults with asthma. Chest.

[17] Tesch F, Sydendal Grand T, Wuestenberg E, Elliott L, Schmitt J, Kuster D (2020). Healthcare costs associated with allergic rhinitis, asthma allergy immunotherapy. Eur. Ann. Allergy Clin. Immunol..

[18] Aumann I, Prenzler A, Welte T, Gillissen A (2014). Epidemiologie und kosten von asthma bronchiale in Deutschland – eine systematische literaturrecherche. Pneumologie.

[19] Valbert F, Neusser S, Riederer C, Wobbe-Ribinski S, Klimek L, Sperl A, Pfaar O, Werfel T, Hamelmann E, Neumann A, Wasem J, Biermann-Stallwitz J (2021). Health care situation in patients with allergic respiratory diseases with special focus on specific immunotherapy. Allergo J. Int..

[20] Bundesministerium für Gesundheit (2019) (Federal Ministry of Health): Gesetzliche Krankenversicherung - Endgültige Rechnungsergebnisse 2018 (KJ1-Statistik)

[21] Bundesministerium für Gesundheit (Federal Ministry of Health) (2019) Gesetzliche Krankenversicherung Leistungsfälle und –tage 2018 (KG2-Statistik)

[22] Bundesministerium für Gesundheit (Federal Ministry of Health) (2018) Gesetzliche Krankenversicherung Endgültige Rechnungsergebnisse 2017 (KJ1-Statistik)

[23] Bundesministerium für Gesundheit (Federal Ministry of Health) (2018) Gesetzliche Krankenversicherung Leistungsfälle und –tage 2017 (KG2-Statistik)

[24] Bundesministerium für Gesundheit (Federal Ministry of Health) (2017) Gesetzliche Krankenversicherung Endgültige Rechnungsergebnisse 2016 (KJ1-Statistik)

[25] Bundesministerium für Gesundheit (Federal Ministry of Health) (2017) Gesetzliche Krankenversicherung Leistungsfälle und –tage 2016 (KG2-Statistik)

[26] Bundesministerium für Gesundheit (Federal Ministry of Health) (2016) Gesetzliche Krankenversicherung Endgültige Rechnungsergebnisse 2015 (KJ1-Statistik)

[27] Bundesministerium für Gesundheit (Federal Ministry of Health) (2016) Gesetzliche Krankenversicherung Leistungsfälle und –tage 2014 (KG2-Statistik)

[28] Bundesministerium für Gesundheit (Federal Ministry of Health) (2016) Gesetzliche Krankenversicherung Leistungsfälle und –tage 2015 (KG2-Statistik)

[29] Bundesministerium für Gesundheit (Federal Ministry of Health) (2015) Gesetzliche Krankenversicherung Endgültige Rechnungsergebnisse 2014 (KJ1-Statistik)

[30] GKV-Spitzenverband (2021) (National Association of Statutory Health Insurance Funds): Kennzahlen der gesetzlichen Krankenversicherung

[31] Ludwig K, Graf von der Schulenburg J-M, Greiner W (2018). German Value Set for the EQ-5D-5L. PharmacoEconomics.

[32] Statistisches Bundesamt: Städte nach Fläche, Bevölkerung und Bevölkerungsdichte am 31.12.2019. https://www.destatis.de/DE/Themen/Laender-Regionen/Regionales/Gemeindeverzeichnis/Administrativ/05-staedte.html (2019). Accessed 2 January 2021

[33] Statistische Ämter des Bundes und der Länder: Personen in Bedarfsgemeinschaften nach Geschlecht, Nationalität, Alter und Erwerbsfähigkeit des Leistungsberechtigten - Stichtag 31.12.2018 - regionale Tiefe: Kreise und kreisfreie Städte. https://www.regionalstatistik.de/genesis/online/data?operation=table&code=22811-02-02-4&levelindex=1&levelid=1603190399759 (2018). Accessed 2 January 2021

[34] Statistische Ämter des Bundes und der Länder: Veräußerungsfälle, veräußerte Fläche, Kaufsumme, durchschnittlicher Kaufwert nach Baulandarten - Jahressumme - regionale Tiefe: Kreise und kreisfreie Städte. https://www.regionalstatistik.de/genesis/online/data?operation=table&code=61511-01-03-4&levelindex=1&levelid=1603190726693 (2018). Accessed 2 January 2021

[35] Heineberg H (2017) Stadtgeographie. utb Geographie, Paderborn

[36] Diekmann A, Meyer R (2010). Demokratischer smog? eine empirische untersuchung zum zusammenhang zwischen sozialschicht und umweltbelastungen. Köln Z Soziol..

[37] von Mutius E, Heinrich J (2020). Schadstoffe und Atemwegserkrankungen.

[38] Rudack C, Sachse F, Jörg S (2003). Aeroallergene von zunehmender bedeutung für die allergische rhinitis. HNO.

[39] Baur X (1999). Berufs- und umweltbedingtes Asthma bronchiale. Internist.

[40] Umweltbundesamt - Fachgebiet "Beurteilung der Luftqualität": Stationsdatenbank des Umweltbundesamtes. https://www.env-it.de/stationen/public/open.do. Accessed 2 January 2021

[41] LAUNIX: Deutsche Postleitzahlen mit Geodaten verknüpft. https://launix.de/launix/launix-gibt-plz-datenbank-frei/ (2015). Accessed 2 January 2021

[42] Canonica G, BaenaCagnani C, Bousquet J, Bousquet J, Lockey R, Malling H-J, Passalacqua G, Potter P, Valovirta E (2007). Recommendations for standardization of clinical trials with allergen specific immunotherapy for respiratory allergy. A statement of a World Allergy Organization (WAO) taskforce. Allergy.

[43] Deutsche Atemwegsliga e.V.: Fragebogen zur Asthmakontrolle - ACT. https://www.atemwegsliga.de/asthmakontrolltest.html (2021). Accessed 3 March 2021

[44] Ferring D (2003). Kurzbeschreibung und forschungsbericht zum “fragebogen zur erfassung gesundheitsbezogener kontrollüberzeugungen” (FEGK).

[45] Valbert F, Neusser S, Pfaar O, Klimek L, Sperl A, Werfel T, Hammelmann E, Riederer C, Wobbe-Ribinski S, Hillerich V, Neumann A, Wasem J, Biermann-Stallwitz J (2022). Care of allergic respiratory diseases in Germany – Predictors and deficits. Clin. Exp. Allergy.

[46] Umweltbundesamt: Luftschadstoffe im Überblick - Feinstaub. https://www.umweltbundesamt.de/themen/luft/luftschadstoffe-im-ueberblick/feinstaub (2021). Accessed 24 April 2021

[47] Umweltbundesamt: Luftschadstoffe im Überblick - Stickstoffoxide. https://www.umweltbundesamt.de/themen/luft/luftschadstoffe-im-ueberblick/stickstoffoxide (2021). Accessed 24 April 2021

[48] Umweltbundesamt: Luftschadstoffe im Überblick - Ozon. https://www.umweltbundesamt.de/themen/luft/luftschadstoffe-im-ueberblick/ozon (2021). Accessed 24 April 2021

[49] Hoffmann F, Andersohn F, Giersiepen K, Scharnetzky E, Garbe E (2008). Validierung von Sekundärdaten. Bundesgesundheitsbl..

[50] Münch C, Gottschall M, Hübsch G, Köberlein-Neu J, Schübel J, Bergmann A, Voigt K (2016). Qualität der hausärztlichen diagnosedokumentation in patientenakten – eine analyse am beispiel von schilddrüsenerkrankungen. Z Evid. Fortbild. Qual. Gesundhwes.

[51] Behrendt C-A, Heidemann F, Rieß HC, Stoberock K, Debus SE (2017). Registry and health insurance claims data in vascular research and quality improvement. Vasa.

[52] Schubert I, Ihle P, Köster I (2010). Interne validierung von diagnosen in GKV-routinedaten: konzeption mit beispielen und falldefinition. Gesundheitswesen.

[53] Schubert I, Köster I, Küpper-Nybelen J, Ihle P (2008). Versorgungsforschung mit GKV-Routinedaten. Bundesgesundheitsbl.

[54] Gansen FM (2018). Health economic evaluations based on routine data in Germany: a systematic review. BMC Health Serv. Res..

[55] Simoens S (2012). The cost-effectiveness of immunotherapy for respiratory allergy: a review. Allergy.

[56] Greiner W, Graf v. d. Schulenburg, JM, Gillissen A. (2003). Kosten und nutzen der hyposensibilisierung bei allergem asthma und Rhinitis. Gesundheitsökonomie Qualitätsmanagement.

[57] Schramm B, Ehlken B, Smala A, Quednau K, Berger K, Nowak D (2003). Cost of illness of atopic asthma and seasonal allergic rhinitis in Germany. Eur. Respir J..

[58] Zuberbier T, Lötvall J, Simoens S, Subramanian S, Church M (2014). Economic burden of inadequate management of allergic diseases in the European union: a GA2LEN review. Allergy.

[59] Grochtdreis T, Dams J, Konig HH, Konnopka A (2019). Health-related quality of life measured with the EQ-5D-5L: estimation of normative index values based on a representative German population sample and value set. Eur. J. Health Econ..

[60] Huber MB, Felix J, Vogelmann M, Leidl R (2017). Health-related quality of life of the general German population in 2015: results from the EQ-5D-5L. Int. J. Environ. Res. Public. Health..

[61] Chen H, Cisternas MG, Katz PP, Omachi TA, Trupin L, Yelin EH, Balmes JR, Blanc PD (2011). Evaluating quality of life in patients with asthma and rhinitis: English adaptation of the rhinasthma questionnaire. Ann Allergy Asthma Immunol..

[62] Szentes BL, Schultz K, Nowak D, Schuler M, Schwarzkopf L (2020). How does the EQ-5D-5L perform in asthma patients compared with an asthma-specific quality of life questionnaire?. BMC Pulmonary Med..

[63] Bousquet J, Arnavielhe S, Bedbrook A, Fonseca J, Morais Almeida M, Todo Bom A, Annesi-Maesano I, Caimmi D, Demoly P, Devillier P, Siroux V, Menditto E, Passalacqua G, Stellato C, Ventura MT, Cruz AA, Sarquis Serpa F, da Silva J, Larenas-Linnemann D, Rodriguez Gonzalez M, Burguete Cabanas MT, Bergmann KC, Keil T, Klimek L, Mosges R, Shamai S, Zuberbier T, Bewick M, Price D, Ryan D, Sheikh A, Anto JM, Mullol J, Valero A, Haahtela T, Valovirta E, Fokkens WJ, Kuna P, Samolinski B, Bindslev-Jensen C, Eller E, Bosnic-Anticevich S, O'Hehir RE, Tomazic PV, Yorgancioglu A, Gemicioglu B, Bachert C, Hellings PW, Kull I, Melen E, Wickman M, van Eerd M, De Vries G (2018). The allergic rhinitis and its impact on asthma (ARIA) score of allergic rhinitis using mobile technology correlates with quality of life: The MASK study. Allergy.

[64] Herdman M, Gudex C, Lloyd A, Janssen M, Kind P, Parkin D, Bonsel G, Badia X (2011). Development and preliminary testing of the new five-level version of EQ-5D (EQ-5D-5L). Qual. Life Res..

[65] Konnopka A, Koenig HH (2017). The “no problems”-problem: an empirical analysis of ceiling effects on the EQ-5D 5L. Qual. Life Res..

[66] Burr H, Kersten N, Kroll L, Hasselhorn HM (2013). Selbstberichteter allgemeiner gesundheitszustand nach beruf und alter in der erwerbsbevölkerung. Bundesgesundheitsblatt - Gesundheitsforschung - Gesundheitsschutz..

[67] Ellert U, Kurth BM (2013). Gesundheitsbezogene lebensqualität bei erwachsenen in Deutschland. Bundesgesundheitsbl..

[68] Oddoy A (2003). Gesundheitliche wirkungen des ozons aus pathophysiologischer sicht.

[69] Hinz A, Klaiberg A, Brähler E, König HH (2006). Der lebensqualitätsfragebogen EQ−5D: modelle und normwerte für die allgemeinbevölkerung. Psychother Psych. Med..

[70] Hinz A, Klaiberg A, Schumacher J, Brähler E (2003). Zur psychometrischen qualität des lebensqualitätsfragebogens nottingham health profile (NHP) in der allgemeinbevölkerung. Psychother Psych. Med..

[71] Barth S, Haas J-P, Schlichtiger J, Molz J, Bisdorff B, Michels H, Hügle B, Radon K (2016). Long-term health-related quality of life in German patients with juvenile idiopathic arthritis in comparison to German general population. PLoS ONE..

